# Robot-Mediated Inclusive Processes in Groups of Children: From Gaze Aversion to Mutual Smiling Gaze

**DOI:** 10.3389/frobt.2022.729146

**Published:** 2022-03-03

**Authors:** Sylvaine Tuncer, Sarah Gillet, Iolanda Leite

**Affiliations:** Royal Institute of Technology (KTH), Stockholm, Sweden

**Keywords:** interactions in groups, robot-mediated interaction, video analysis, gaze behaviour, conversation analysis, ingroup inclusion, interdisciplinary study

## Abstract

Our work is motivated by the idea that social robots can help inclusive processes in groups of children, focusing on the case of children who have newly arrived from a foreign country and their peers at school. Building on an initial study where we tested different robot behaviours and recorded children’s interactions mediated by a robot in a game, we present in this paper the findings from a subsequent analysis of the same video data drawing from ethnomethodology and conversation analysis. We describe how this approach differs from predominantly quantitative video analysis in HRI; how mutual gaze appeared as a challenging interactional accomplishment between unacquainted children, and why we focused on this phenomenon. We identify two situations and trajectories in which children make eye contact: asking for or giving instructions, and sharing an emotional reaction. Based on detailed analyses of a selection of extracts in the empirical section, we describe patterns and discuss the links between the different situations and trajectories, and relationship building. Our findings inform HRI and robot design by identifying complex interactional accomplishments between two children, as well as group dynamics which support these interactions. We argue that social robots should be able to perceive such phenomena in order to better support inclusion of outgroup children. Lastly, by explaining how we combined approaches and showing how they build on each other, we also hope to demonstrate the value of interdisciplinary research, and encourage it.

## Introduction

In many countries worldwide, schools take in children who recently arrived from abroad with their family. It is often the case that these children master neither the language nor various local customs. This can lead to feelings of limited shared background with the other children, and, in turn, difficulties to communicate and socialise at school. Both for children’s wellbeing and for society as a whole, it is therefore important to find ways and means of including newly-arrived children in the various social spheres and activities. This paper results from an interdisciplinary collaboration aiming to propose ways of supporting the inclusion of newly-arrived children at school, through a social robot. The contribution is twofold. Firstly, we present the findings from qualitative, fine-grained analyses of video-recorded interactions between a robot and children, data that were previously collected and used for quantitative analysis in a social robotics study. The findings presented are interactional processes of inclusion in groups of children through the robot as a mediator. Secondly, we describe and reflect on the research process, initially aiming to explore the potentials of interdisciplinary research between social robotics and research on interactions in the social sciences. Despite the shared reliance on video-recorded, face-to-face interactions to study interactional phenomena, and a shared interest in the use of space and coordination through gaze for example, the connections or rather actual collaboration between HRI and interactional research remain surprisingly rare. We hope to demonstrate that such interdisciplinary collaboration can indeed benefit both fields by informing social robotics and design, and bringing new knowledge of social interactions and interactional phenomena. From this, we propose a guideline to undertake interdisciplinary research, and hope to encourage such enterprises.

Processes of inclusion and exclusion from groups have been extensively studied in social psychology, with the concepts of ingroup and outgroup referring to statuses of belonging. Studies focusing on how people can categorise others as outgroup have shown that categorisation tends to be grounded in first impressions, largely on visual cues (Abrams et al., 2005), and that children start to categorise at a very early age (Aboud, 2003). Because being part of the ingroup “leads to feelings of loyalty and a perception that one’s teammates are superior to those on the other team” (Nass and Moon 2000, p.87), there is a tendency for ingroup members to exclude outgroup individual(s). Such behaviours have obvious negative consequences on communities and society broadly, such as division and conflict. By identifying and bringing new knowledge of these banal and yet detrimental processes in groups of children, our study has the potential to help children and educators to subvert them.

Our original study is based on the premise that newly-arrived children are outgroup members. As we explain in more detail in the “Materials” section, newly-arrived children went in a separate class and could therefore be considered as “outgroup”, and yet they did not all have difficulties interacting with the ingroup children. Therefore, for this study, we considered how and if children interacted with each other in addition to the notion of ingroup or outgroup. Additionally, to highlight the specific relationship between two children in the analyses of video extracts, in this article we refer to the pair formed by the outgroup and one of the two ingroup children as “unacquainted children”.

The initial motivation of our original study was to design a social robot that would foster the inclusion of a newly arrived child in groups of children in their school while playing a board game together. The aim was not the interaction between the robot and the children, rather the interactions between the children, through the robot as a mediator (Verbeek, 2015). First, we implemented a study in the form of a typical HRI protocol (Hoffman and Zhao 2020). We designed a social robot behaviour and implemented this behaviour on a Cozmo robot. Then we tested the robot behaviour with children, we video-recorded the experimental sessions, collected a set of quantitative data, and analysed the computed game measures. With quantitative results, we showed in what ways this robot behaviour was effective to foster participation and collaboration in groups of children involving an outgroup child (see Gillet et al., 2020; Gillet and Leite 2020).

In an endeavour to develop an interdisciplinary perspective on the matter, we undertook a second study bringing in a distinctive and complementary approach from the social sciences. We interrogated the same set of video data anew in the perspective of Ethnomethodology and Conversation Analysis (thereafter EMCA) (Garfinkel 1967; Sidnell and Stivers 2012). EMCA aims to unpack and show the organisation and unfolding of interactions on a turn-by-turn basis, and how actions—turns-at-talk, embodied actions or complex multimodal moves—are produced and recognised as performing meaningful social actions. Discarding the micro/macro divide, this approach shows that social structures, norms, or moral order, which are conventionally seen as imposed on situations and actors, are actually accomplished in everyday interactions, be they face-to-face or mediated. Another central characteristic of this approach is that it takes the participants’ perspective and aims to understand their local, practical problems. From this viewpoint, EMCA then seeks to identify typical forms of actions which participants produce and recognise as interactionally meaningful and making relevant a set of potential next actions, to progress the course of actions. But because they are so ordinary, these practices remain “seen but unnoticed” most of the time. They can become visible in audio- or video-recordings, viewed repeatedly and analysed qualitatively, in great detail. Unlike psychology, which largely relies on experimental data, EMCA generally uses recordings of naturally occurring interactions as data, that is, interactions which would have taken place had the study not been conducted, thus not occasioned by researchers. In addition to being qualitative and taking the participants’ perspective, this approach is also unmotivated in the sense that the research questions and focus of a study are partly defined at the start but also largely revised in the course of the analyses.

Thus, the present article results mainly from this second, extensive round of qualitative analysis of the video recordings of children interacting in groups and with the robot as a mediator, drawing on EMCA. In our analysis we pay particular attention to the sequential organisation and the quality of the children’s and the robot’s actions. We examine their embodied, multimodal conduct including features such as gaze, gesture or spatial orientation, as well as talk, even though our data involve very little talk. We identify, describe and draw the implications of patterns and practices through which forms of interaction were (co-)produced. We study the ways in which the children respond, or attempt to respond, to the conduct of the robot, and seek to communicate and initiate actions with each other. We identify and unpack a set of commonplace interactional phenomena, which we argue are among those which social robots should be able to detect and respond to, to foster inclusion in groups of children, but also more broadly to become competent interactional partners in the future. This second, qualitative study thus (1) complements our first study based on task measures by showing how the robot (and the game) can encourage unacquainted children to make contact; (2) informs robot design by drawing attention to the complexities of multiparty interactions in naturalistic environments which social robots should be able to deal with in the future; and (3) contributes to interactional research on gaze and children interactions. Lastly, (4) by describing how we applied two different approaches and methods to the data, we hope to provide a guideline for one type of interdisciplinary research, and to encourage researchers to engage in such projects.

## Background

### Learning From Human-Robot Interactions “in the Wild”

There is growing awareness in HRI research that in order to improve social robots and enable broader adoption in the future, we need to bring them outside laboratories and experiments, in order to observe their interactions with humans in uncontrolled, “real-life” situations (e.g., Jung and Hinds 2018; Park et al., 2020; Fischer 2021). The challenges for design are considerable: it demands from the robot a capacity to perceive and interpret the “signals” it receives from the environment, to respond appropriately, and to understand and adapt on a moment-to-moment basis to a continuously changing situation (Breazeal 2003). Firstly, “signals” from humans are most often complex multimodal gestalts (Mondada 2014), involving language, bodily conduct and orientation to the environment. They also gain their meaning and import from various aspects of the situation: the setting, the larger course of action, participants’ relationship, their practical aims, etc. Rather than mere transmission of information, they are complex social actions. The temporal dimension is also central as the progression of the interaction and the evolution of the context imbue each action with particular meaning and import. Natural interactions in the wild demand high-level perceptual, cognitive and social competences often beyond the capacities of today’s robots. A clearer view of the requirements is needed to work towards such capacities.

Non-experimental observational studies let us see not only how robots understand, respond and adapt to human conduct, but also how we, humans, respond to them, how we consider them—what sort of agents or entities, for example insensitive machines or beings with moral rights, capable of feeling emotions. In short, some issues which are critical for HRI become uniquely amenable to research with naturalistic observational studies.

### Group Interaction in HRI

Many interactions in the real world involve more than two parties. In the HRI community, there has been a growing interest in the latter decade or so to study robots in groups (see Strokhorb et al., 2020). Prior literature has investigated more specifically how a robot can benefit a group by shaping its dynamics (e.g., Pitsch et al., 2017; Strohkorb et al., 2018; Jung et al., 2020; among children in particular; Lemaignan et al., 2018). Some have studied how they can attribute roles and facilitate intimacy in interactions. For example, Mutlu et al. (2009) show that a robot’s gaze can influence people’s conversational roles. More recently, Strohkorb et al., 2018 show that robots can increase engagement and trust-related behaviours between team members who are making vulnerable statements. Utami and Bickmore (2019) show how a robotic therapist can improve intimacy and positive affect in romantic couples. Robots have also been used as mediators in conflict situations. For example, a robot can promote more constructive conflict solving behaviour in case of object possession conflicts among children (Shen et al., 2018). Or, when personal violations induce a group conflict, a robot acting as an emotional regulator can help to regulate and call attention to a conflict (Jung et al., 2015).

Moreover, robots are shown to facilitate collaboration. One work shows that a robot in a moderator role can influence perceived group cohesion by addressing certain participants more often (Short and Mataric, 2017). Another work uses a microphone-shaped robot, which can balance the conversation of a group of three and thereby achieve higher group performance (Tennent et al., 2019). Similarly, Gillet et al. (2021) explored how gaze can lead to more balanced participation. The robot can encourage passive members to participate more actively with non-verbal and indirect cues. When improving human-human collaboration among children, relation-reinforcing utterances could enhance the perception of team performance (Strohkorb et al., 2016). Similarly, Shimada et al. (2012) show how a robot can increase motivation through relationship-building and encourage behaviours in a Lego-building task. Particularly relevant to our study, Strohkorb Sebo et al. (2020) also aim to foster inclusion in groups, with a robot providing verbal encouragement for outgroup members to participate more, with different strategies tested in the study. While the robot’s verbal encouragements prove effective, another, less positive finding is that this robot behaviour also suppressed ingroup members’ efforts to include the outgroup member, suggesting detrimental effects on human sociality itself. Lastly, Fraune et al. (2019), studying a robot’s interactions with groups of humans *in the wild*, raise a crucial, often overlooked dimension of interactions in groups: interactions are shaped by group characteristics and norms, which therefore need to be better understood and integrated in robot design.

### Conversational-Analytic Research in HRI

The contribution of EMCA to HRI research is far from new, shedding light on interactional phenomena relevant to robot designers. For example, Yamazaki et al. (2012) analysed the practices of human museum guides in order to implement similar practices in a robot guide, which the authors showed successfully improved visitors’ engagement. Pitsch et al. (2013) show how human tutors adapt their demonstrations to a robot’s feedback as it follows their manual actions, and thereby suggest ways in which robots could generate feedback from human interlocutors and obtain input to shape their own actions. Or, Pelikan et al. (2020) show how human participants (household members) make sense of a social robot’s displays of emotions. Our study builds on the much broader existing work briefly exemplified here, by exploring how the conduct of the robot can influence the moment-to-moment enfolding of communication among children, foster the inclusion of an outgroup child, and support a process of group formation. The ethnomethodological, conversation-analytic approach to interaction has been used to understand in particular the use of space, spatial formation behaviour, and the reliance on gaze to coordinate mutual involvement in human-robot interactions.

### Gaze and Children Laughter in Conversation Analysis

Making first contact with strangers requires interactional work (e.g., Pillet-Shore 2011), even when the stranger is categorised as a peer member of society and a large common ground (Clark 2006) is assumed. While adults can rely on social skills and methods to do so out of their experience, children are somehow left to their own devices. Since Kendon (1967) and related pioneering works on gaze, it has been largely established that participants spend a significant amount of time looking at each other’s face when they interact. Conversation Analysis began to study gaze early on as a central feature of face-to-face interactions (Goodwin 1980; Heath 1984). Subsequently, gaze has been extensively studied as a central resource to manage participation and engagement (for an overview, see Rossano 2013). Another approach has been emerging recently, investigating gaze in action formation (Stivers and Rossano 2012; Streeck 2014), either as part and parcel of a multimodal action such as a question pursuing an answer, or as an action in and of itself. Coincidentally, the latter has been mostly investigated in interactions between children and adults, showing for example how infants looking persistently towards a caregiver are understood and responded to as calling for assistance, as understanding that they are approaching, or as searching them out (Kidwell 2009). Our findings build on both aspects of gaze in interaction: in *Robot Requires to Take Action: Mutual Gaze as Part of Giving and Asking for Instructions* (mutual) gaze plays an essential role in asking for and producing instructions; and in *The Robot’s Conduct Elicits Emotional Reactions: Looking and Smiling to the Other as Sharing Emotions (Towards Bonding)*, mutual gaze is a social action and achievement whereby the unacquainted children share an emotional reaction and make (positive) contact.

Focusing on children’s laughter and emotion sharing in preschool, Cekaite and Andren (2019) show that children primarily seek and receive affiliation through laughter in the peer group. They emphasise that emotion and norm sharedness in social interaction are not just a matter of communicating an emotion from one person to another, but an intricate process of inviting the others into or negotiating the common emotional and experiential ground. Looking similarly at interactions among children, Strid and Cekaite (2021) focus on how children calibrate their emotional stances and affiliation in sequences of shared laughter. The establishment of mutual gaze is shown to be important in initiating, reciprocating and sustaining shared emotional stance through the performance of laughter. Our study builds on the former by showing not only that similar processes of sharing emotions and laughing together can be vehicles for unacquainted children to make contact, but also how a robot mediator can be particularly helpful to facilitate such processes by providing a joint focus and creating opportunities for interaction.

## Materials and Method

At the start of this project, we developed a game involving a social robot programmed to support inclusion and collaboration in groups of children. In the game, children had to move coloured cubes on a board. The robot aimed to encourage the least involved child to participate more, and all the children to move their cubes in each other’s proximal space as a way of making contact. To then test the robot design, we set up an experiment with children in a local school with the help of teachers.

Our study involved, in total, 39 children, 21 male and 18 female, participated in the initial experiment with a mean age of 10.46 years (SD = 0.67). All children were going to the same school but to different classes. Indeed, in the school where we conducted the experiment, newly-arrived children were in a special class where they focused on language acquisition. They were coming from Asia (6), South America (2), and other parts of Europe, and they had been living in Sweden from 0.5 up to 24 months (M = 10.5, SD = 8.71). For the experiment, Children were recruited with the help of teachers who distributed and collected information sheets and consent forms. The initial study was ethically approved by the Swedish Ethical Review Board (Dnr 2019-05085).

**FIGURE 1:**
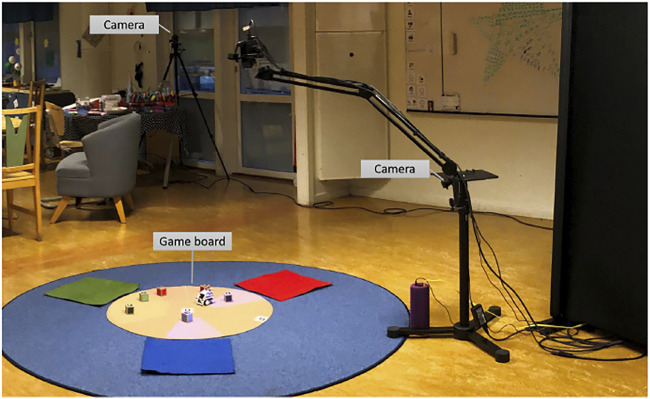
Experimental set-up used during the study. Each child was seated on one of the colourful patches around the game board. The sessions were filmed from both cameras.

Groups of three children were formed, including one newly-arrived child and two children who had grown up in the country, or had resided there for a long time. Thirteen groups of children were video-recorded playing the game. The children were seated in a circle on the floor (Figure 1) and invited to participate in a joint activity focusing on an animated, non-anthropomorphic robot (Cozmo).

Each group participated in the experiment twice, each session taking place 1 week apart. Each session was divided in two: a pre-game phase where children were left to discover how the robot behaved; and the game phase, where the children tried to complete the game by moving cubes on the board supported by the robot. In both phases, the robot behaviour was augmented by projected lights. The pre-game phase served to introduce children to the robot’s conduct and how it asked to “replace cubes”. Children received one cube each and the robot was introduced as “showing them what they could do with the cubes”. No other information about the robot’s role or capability was given. After the children completed the pre-game phase, the game was explained. The robot stayed on the board and was not further mentioned. During the game phase, the robot was sometimes helping to complete the game but mainly prompting children to participate and place their cubes more often into each other’s proximal spaces. The game was completed when a specific arrangement of cubes on the board was achieved.

In previous publications (e.g., Gillet et al., 2020), we studied how actively the children played the game, and how much they reached out into one another’s proximal domain, based on their movements. We also compared the adaptive vs. random robot behaviour conditions, and we further analysed the robot’s autonomous encouragements. We showed that the robot appeared successful in perceiving the group’s dynamic, and that children took more initiatives through the adaptive robot behaviour, towards inclusion and collaboration.

For the present paper, we took a fresh, new look at the video recordings, taking a conversation-analytic approach involving systematic, in-depth qualitative analyses. In exploring the data, we focused on the interactions between the robot and the children as they emerged, and did not consider the original study conditions. In order to remain open to what the data would offer, and at the same time inform the initial research questions and goals (testing the robot’s effectiveness in fostering the inclusion of newly arrived children), we looked for (1) if and how the unacquainted children interacted with each other; and (2) if and how the robot and the game mediated or facilitated these interactions. Then, in the sequences where (1) and (2) occurred, we studied (3) how group dynamics were involved and affected.

Upon arrival in the experimental room, the children were asked about their age, which class they were in, and if the two long-residents and the newly-arrived hat met before. Only one of the thirteen groups indicated that they had met before. However, even if a newly-arrived child was included in each of the groups and children had not met before, on viewing the recordings we could see that in some of the groups all three children established eye-contact smoothly from the start. We therefore focused the analyses on four groups out of thirteen where the outgroup child did not participate in the other two children’s interactions. The details about the remaining children are given in Table 1.

**TABLE 1:** Demographics of the participants included in this study.

Group number	Age	Gender	Continent recently arrived from	Time spent in Sweden
1	10, 9, 10	M, M, M	Europe	3 months
2	10, 9, 10	M, M, F	Asia	3 months
5	10, 10, 10	F, F, F	Asia	10 months
12	11, 11, 11	M, F, M	Asia	0.5 months
13	11, 11, 11	M, F, M	South America	18 months

The final data set amounts to 2 h and 50 min of video. In this dataset, gaze appears particularly sensitive among the outgroup and ingroup children. While they acknowledge each other’s presence and display some interest by intermittently looking towards one another, they also predominantly avoid making proper eye contact by averting their gaze before or just as the other returns the look. They unproblematically focus together on the robot and cooperate to play the game, but making and sustaining eye contact appears otherwise difficult. That they avoid each other’s gaze does not mean at all that they ignore each other: more or less frequently they look at each other, but in ways that seem shaped to avoid attracting a look in return^1^. And when mutual gaze occurs, the prompt gaze aversion that follows suggests it was “accidental”.

Thus, we trimmed this corpus looking for instances where unacquainted children made eye contact. We gathered a collection of twenty-two (22) instances which we analysed in detail, with the approach and methods of EMCA explained above. We found that mutual gaze occurs in two distinctive trajectories or sequences of actions, and therefore classified the instances in two groups. In the game phase, the need to take action in the game (partly indicated by the robot) can lead children to look at each other and make eye contact in order to ask for help or produce an instruction; whereas in the pre-game phase, the children can react emotionally to the robot’s conduct and attempt to share emotions by looking at each other and making eye contact. We systematically analysed these two collections, focusing on the sequential emergence of mutual gaze, the robot’s role in this, and the interactional and relational outcomes of these sequences.

## Results

In what follows, we present the findings through detailed analyses of five representative instances where mutual gaze emerges, to show *how* the robot and the game can lead children to interact through gaze. In *Robot Requires to Take Action: Mutual Gaze as Part of Giving and Asking for Instructions*, we focus on instances where interaction serves collaboration to move the game forward, and in *The Robot’s Conduct Elicits Emotional Reactions: Looking and Smiling to the Other as Sharing Emotions (Towards Bonding)* on instances where the children share an emotional reaction to the robot’s conduct. The video extracts are transcribed in the form of image captions and lines of talk (the latter only in Extract 4) placed along a vertical timeline. The numbers on the timeline indicate the time elapsed in seconds (e.g., “0.8” means eight tenths of a second) since the beginning of the clip and the following image or line of talk.

Two characteristics of the sessions from which these video clips are extracted need to be kept in mind. Firstly, it is visible in the recordings that the two ingroup children know each other quite well as they interact like friends, whereas “unacquaintanceship” with the outgroup child is equally observable. Secondly, the outgroup and the two ingroup children predominantly avoid looking at each other, so that the instances we present here—and mutual gazes therein—must be seen as extraordinary.

### Robot Requires to Take Action: Mutual Gaze as Part of Giving and Asking for Instructions

In this section, we focus on mutual gazes which occur during the second phase of the experiment, where the children are invited to play the game. Supported by changes in music and lighting, the robot instructed them to move the cubes to different places on the board. We found that unacquainted children can collaborate to make sense of the instructions. Whether they assume a lack of common language, or simply thereby avoid speaking like they avoid making eye contact, they talk very little if at all, and rely mainly on visual conduct. Therefore, in the production-recognition of embodied instructions or requests for instructions, they massively rely on gaze, on the coordination of looks (at one another and at the material environment), facial expressions and movements, in ways that sometimes lead to mutual gaze.

Extract 1 takes place at the beginning of the game. While they follow the instructions and play, the children are also still figuring out how to understand these instructions and how to make sense of the robot’s conduct. In the few seconds prior to the transcript, the children have been moving several cubes on the board hesitantly, unsure of each of their moves. In what follows, as the outgroup girl on the left-hand side on the images we call Menisha^2^, is visibly about to move a cube, she initiates an interaction with Lisa, asking her through visible, embodied conduct to confirm that the move she is about to make is the right one.

**EXTRACT 1:**
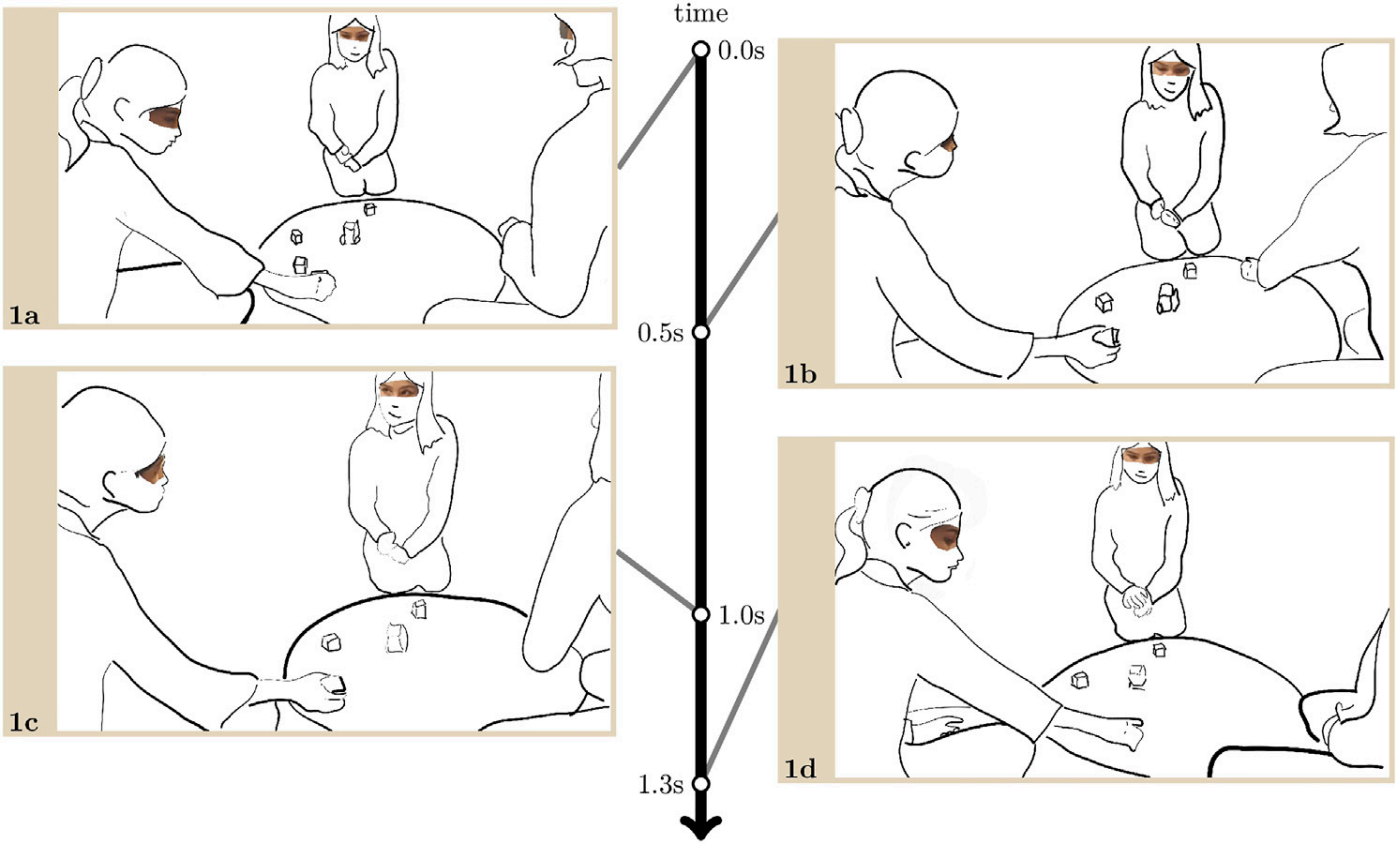


As she is about to place a cube in an empty space (1. a), Menisha withdraws her arm, raises her head and looks at Lisa (1. b), who responds with a series of quick nods before raising her head and looking at Menisha. A brief mutual gaze occurs (1. c), after which Menisha immediately looks towards the board again and places the cube (1. d).

Gaze is central for the two girls to make sense together of the robot’s conduct or rather the absence of the robot’s conduct and agree on the next move to make. Menisha makes her move intelligible as asking for confirmation by looking at Lisa just before putting down her cube. By looking towards Menisha after nodding, Lisa occasions mutual gaze and thereby completes and closes the question-answer sequence. Through this brief interaction, they have reached a shared understanding of the action requested by the robot.

This extract is representative of the type of interactions that the unacquainted children initiated in the game phase. That the girls keep neutral faces throughout suggests that collaborating to make sense of the robot’s instructions and progress the game does not require displaying or pursuing an interpersonal affective stance.

In Extract 2, however, the outgroup child not only initiates the interaction to give an (unsolicited) instruction, he also smiles at his addressee. Just before the extract starts, the robot prompted a cube placement on an empty space of the board by moving towards that space, with a circle of light appearing just in front of the robot to highlight the meaning of its movement. Andrej (middle on the images) is looking towards it, whereas Ali and Erik, the two ingroup children, seem to look at no point in particular on the board (1. a).

**EXTRACT 2:**
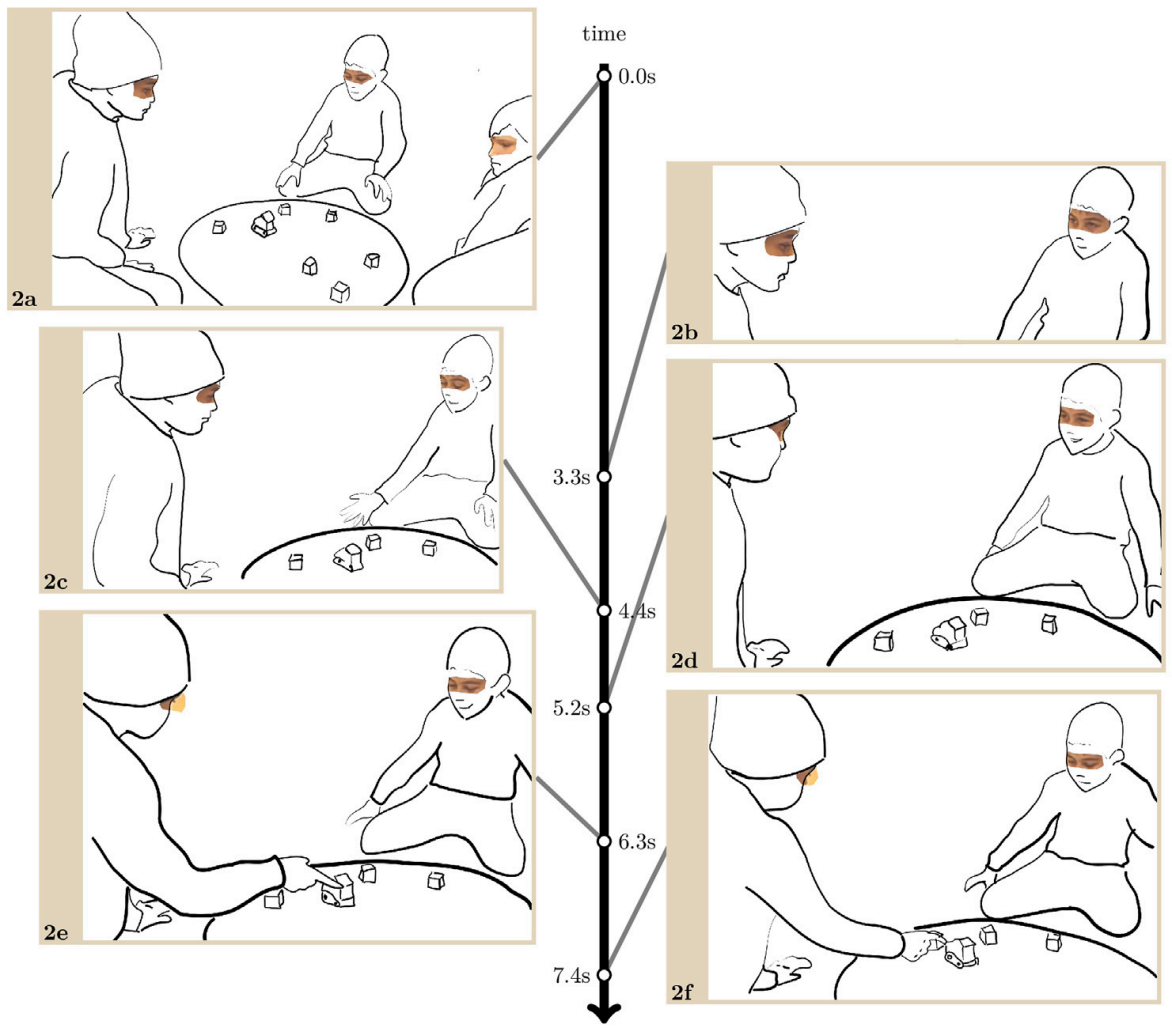


About 3 s after the robot’s prompt, Andrej looks towards Ali (2. b). He does not pursue or await a return look. He looks down to the circle of light again which indicates the exact position the robot wants the cube to be placed in, and points with his right arm towards the cube near the robot. Once Ali has followed Andrej’s pointing gesture with his gaze, Andrej opens his mouth without making sounds, and makes several beating gestures towards one of the cubes with his right hand palm open (2. c). Using embodied conduct only, he is instructing Ali to move the cube, which also implies that Ali is not following or not understanding the robot’s instructions (also supported by the lighting). When he withdraws his arm, Andrej looks towards Ali again and smiles. The smile clearly displays a benevolent attitude towards Ali, which probably aims in part to soften the otherwise authoritative character of his previous action.

While Andrej is still looking and smiling at him, Ali looks at him in turn, but he does not reciprocate the smile (2. d). He shakes his head sideways, thus rejecting Andrej’s instruction and disagreeing with Andrej’s understanding of the robot’s conduct and the game as a whole. Indeed, while the robot is encouraging to move a cube, as it always does, it is not taking into account how close the children are to completing the game. Ali has understood that they had better not move this cube there, but Andrej hasn’t. This is what Ali subsequently explains to Andrej through his embodied conduct: he points to the centre of the board (2. e) where concentric circles display the progression in the game, then moves the cube following Andrej’s instruction (2. f) for Andrej to see that this move takes them backward in the game. Andrej follows with his gaze and progressively relinquishes his smile. Ali then places the cube back, and he moves another one (not reproduced in the transcript).

To sum up, Andrej initiates an interaction with Ali to give an instruction based on his understanding of what the robot is asking to do. As he looks at Ali, he displays a friendly stance by smiling. This attempt to make positive contact, occasioned by collaboration in the game, does not generate affiliation because Ali disagrees with Andrej’s understanding of the game, and for his disagreement to be intelligible as such he does not reciprocate the smile. In other words, Ali keeping a neutral face progresses the course of action and collaboration, even though from an interpersonal perspective Andrej’s smile makes a responsive smile relevant, if not preferred^3^.

In this section, we showed with two contrastive instances that in order to collaborate and move the game forward, children can look at each other and make eye contact. As the robot requests an action from one particular child, and the action is either unclear or not produced, one child can take a step toward another child to give or ask for an instruction, and in order to do this, look at her or him. Considering their gaze behaviour throughout the sessions, this is a remarkable achievement in itself. These looks are immediately recognised as part of action formation in relation to the game, and key to collaboration, especially in the absence of verbal language.

In these collaborative sequences where not only gaze but mutual gaze are central resources, children are focused primarily on moving the game forward. We showed that collaboration can take precedence over displays of interpersonal stances, such as smiling to the other, and therefore, we argue, on relationship building. On the other hand, that collaboration provides the children with opportunities to make contact without taking a particular affective stance towards each other, allows children to communicate precisely because in this situation it does not require personal, affective involvement. If interactions to give or ask for instructions are likely to remain functional, they can also sometimes extend to interpersonal relationship building: as shown with Extract 2, unacquainted children can take a positive stance towards one another. In other words, collaborating to move the game forward, and interacting interpersonally, can either coincide or interfere as two parallel interactional engines.

For the design of social robots, this suggests that a robot with the capacity to perceive mutual gazes between humans in groups, and—more challenging—to recognise emerging forms of collaboration of which these mutual gazes are a part, would be able build on initial instances and encourage the same participants to collaborate again shortly after. Or, again on perceiving emergent collaboration, it could take a different strategy drawn from the phenomena we explore in the next section: attempt to bring in a more interpersonal, affective dimension by fostering an emotional reaction.

### The Robot’s Conduct Elicits Emotional Reactions: Looking and Smiling to the Other as Sharing Emotions (Towards Bonding)

In this section, we focus on mutual gazes between unacquainted children as part of sharing emotions. In the pre-game phase, they frequently smile, laugh, or make facial expressions or sounds, in response to actions from the robot. They seem amused, surprised, or moved by the robot. In HRI, while it is widely acknowledged that people often find robots cute (Gn 2017), whether robots should be designed to be found cute is debated as either necessary for the adoption of social robots in the home (Breazeal 2003) or as an ethically problematic way of deceiving users (e.g., Lacey and Caudwell 2018). In our data, when children found the robot cute or were amused by it, they often attempted to share this emotion by looking at each other, which sometimes led to mutual gaze between the unacquainted children. In many cases, gazes were synchronised so that they were no longer smiling when their eyes met, but in a handful of cases *mutual smiling gazes* occurred, a brief but also highly affiliative interchange. Building on Strid and Cekaite (2021) who focus on children laughing together, we argue that mutual gaze and smile in reciprocating an emotional stance can critically contribute to establishing affiliation and bonding.

Extracts 3 involves the same group of children as Extract 1. Just before the transcript, the robot makes a typical long, singing vocalisation which Lisa reacts to with a smile and audible outbreath hearable as laughter.

**EXTRACT 3:**
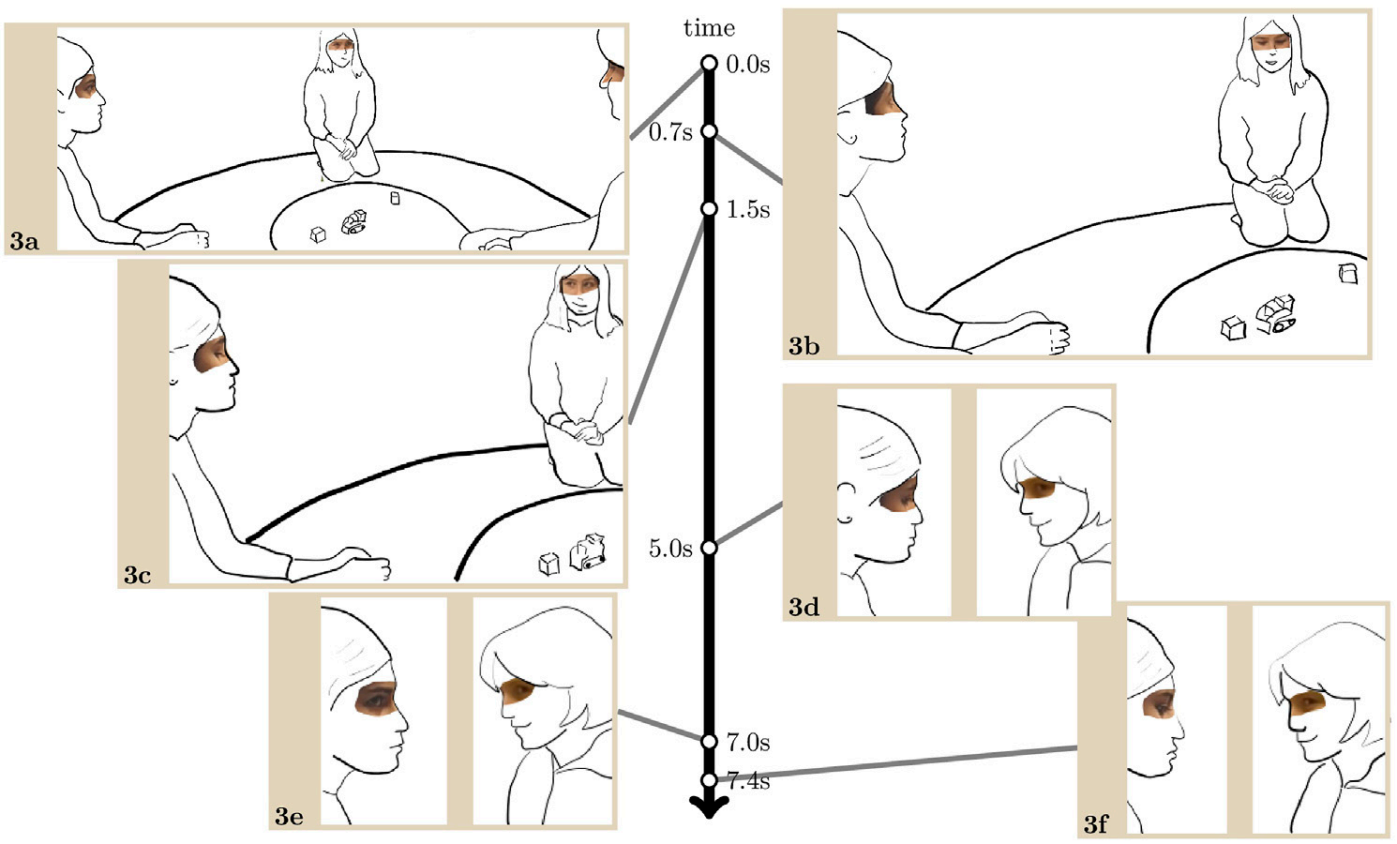


With the smile still on her face, Lisa looks at her friend Gil who is then also smiling, and in the meantime, Menisha briefly glances at Lisa (3. a). When Lisa is looking down at the robot again, Menisha turns and looks at her in a way that is visible to the other participants (3. b). Lisa, with a smile still on her face, reciprocates the gaze very shortly after, but Menisha is already looking down again (3. c). So far, Menisha and Lisa have been taking turns in looking towards each other, missing, or avoiding, each other’s gaze. This attempt to make contact, following and building on the two ingroup children sharing an emotional reaction, embodies an emerging group dynamic.

About 3 s later, Gil attempts to build on this first exchange between Lisa and Menisha by looking and smiling at Menisha (3. d). Two seconds later, Menisha eventually returns Gil’s look too (3. e). This relatively long delay exhibits her reluctance to let mutual gaze occur. As mutual gaze eventually occurs, she does not return Gil’s smile, and looks down again shortly after (3. f).

This extract is typical of a number of instances in our data where the children react to the robot’s conduct with displays of emotions which they share by looking towards one another. This can be done in either one or two steps, first reacting individually and then turning to the other; or turning to the other while reacting. Public looks, that is, looks which are produced so as to be seen by others, are generally responded to, which provides further evidence that children’s glances to one another are interactions in and as themselves. They rarely coincide in a mutual gaze mainly because they tend to be very brief.

Children returning the look with delay, and thus the very rare occurrence of mutual gaze, reflect, rather, the very sensitive nature of eye contact between strangers, even when the other party displays a highly positive stance by smiling. In Extract 3, Menisha appears simply scared of treading unknown territories by returning Gil’s gaze and smile.

Children can also be more confident and let mutual gaze occur as they share their emotional reaction to the robot’s conduct. Still, some emotional reactions are less clear and straightforward to align with than laughter. In addition to timing and coordinating looks, this can leave children with the interactional challenge of appropriately aligning to the other’s stance. In Extract 4, Malin and Ben are the ingroup children, Tim the outgroup child. Just prior to the transcript, Malin placed a cube in front of the robot, and the latter fell backwards by shaking its arms above and hitting the cube. The transcript starts as Malin suddenly recoils, brings her hands to her face (4. a) and produces a response cry, “*oh*”: she reacts to the robot falling as a surprise, but also as an accident, unintended and potentially harmful for the robot.

**EXTRACT 4:**
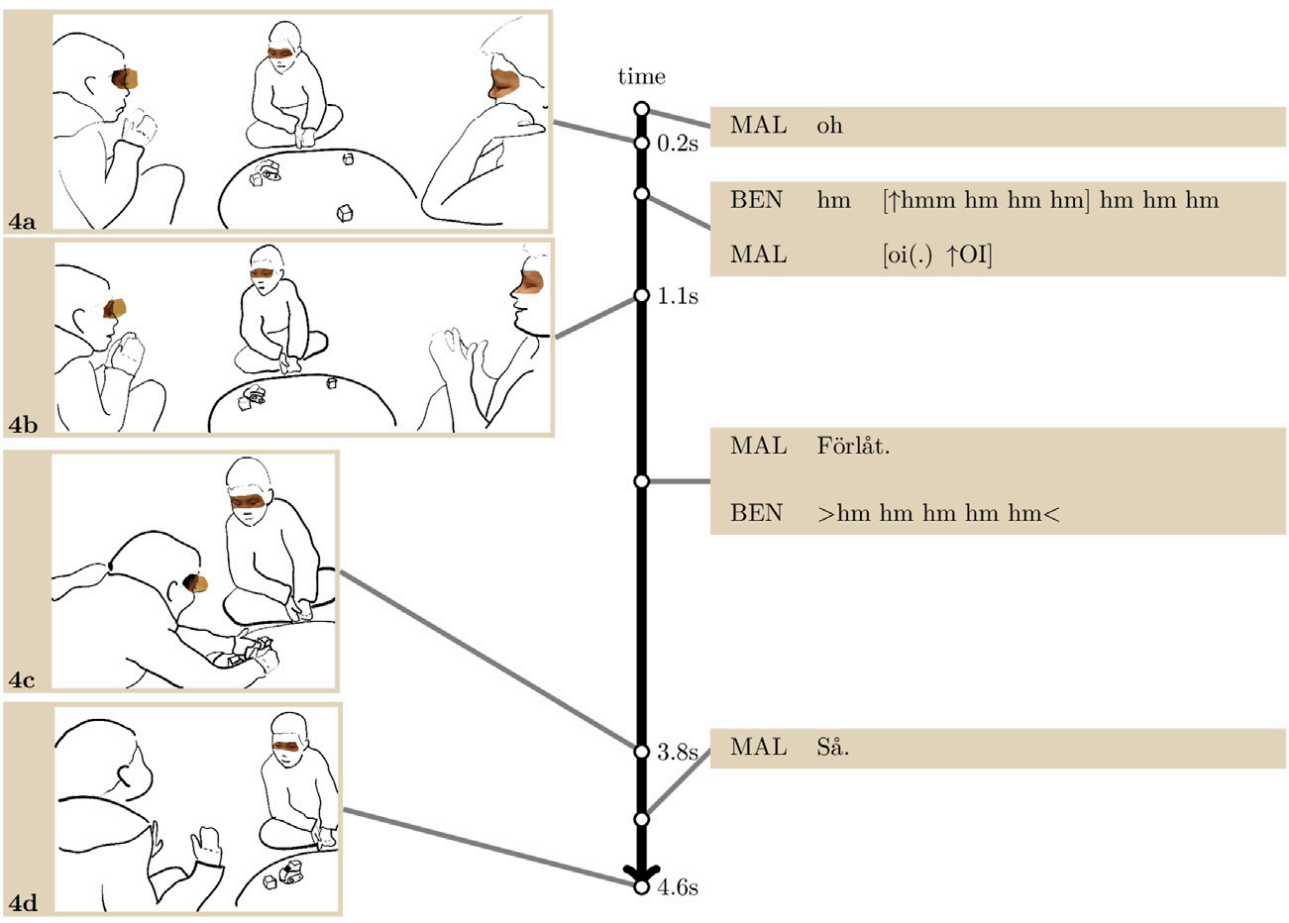


Shortly after Malin’s reaction, Ben (right on the images) giggles in high-pitched, rapid exhalations (line 2). Thus, he responds to the robot’s conduct as laughable, rather than as a potentially serious incident like Malin, which counterbalances her reaction. Tim, on the other hand, aligns with Malin’s reaction by also recoiling and making an O shape with his mouth as Malin produces a second response cry (“*oi*”, line 3). He looks briefly towards her (4. b), and then as she bends down to the robot, he follows her movements with his gaze, and takes a neutral face again. Malin initiates a remedial interchange with the robot: while bending towards it, she apologises (“*förlåt.*“, line 4, “sorry” in Swedish) and puts it back on its wheels (4. c). A remedial interchange re-establishes the equilibrium of moral order when a normative transgression has been committed; it transforms “what could be seen as offensive into what can be seen as acceptable” (Goffman 1971: 109). Here, Malin shows that she is responsible for having made the robot fall, that she has caused some harm, and is morally obliged to the robot to repair the harm. In other words, she treats the robot like a subject with moral rights.

Once she has put the robot back on its wheels, she moves her upper body back in a straight position, and meanwhile she looks at Tim with a smile. While looking at him, she says “°*så*°” (“*this way*” in Swedish, line 7) and places her hands palm open upwards (4. d): she shows to Tim that she has repaired the offense. Tim returns the look, but he does not smile (4. d), and they both look down again shortly after.

In this instance, the children react to the robot shaking its arms, vocalising and falling backwards as either laughable (Tim) or on the contrary as an accident caused by Malin’s prior action, with the robot somehow a victim. The outgroup child makes the first attempt to share emotions by looking at Malin (4. b), but at this point Malin is preoccupied with remedying the harm done to the robot. The robot remains the centre of attention especially as Malin engages in a remedial interchange with it. Once her remedial action is complete, she builds on Tim’s attempt by looking (back) at him with a smile, addressing him as a witness of that interchange. However, at this point, Tim is *visually* following Malin’s actions with the robot, but not the *meaning* she is giving to them, and he does not respond with an affiliating smile.

Ben’s audible giggles support the exchange between Tim and Malin, first after the robot falls, then after Malin apologises. These are pivotal moments to cast the prior actions and the situation as funny, either the robot falling or Malin’s reaction itself, treating the robot as a victim. A group dynamic thus emerges and provides a supportive background for Malin and Tim to make contact and interact.

With Extracts 3, 4, we showed how unacquainted children can look at each other, sometimes with a smile, as they either laugh or display surprise or distress in response to an action from the robot. We emphasised the many contingencies at play in these elusive choreographies of looks and smiles, so that mutual gaze rarely happens, even less coinciding with aligned emotional displays and displays of interpersonal stances.

Extract 5, taken from the second session between the children from Extract 4 ^4^, is one of the few instances where the unacquainted children look *and* smile at each other. They have spent 2 min with the robot, mainly observing its actions so far. A few seconds before the transcript starts, Tim moved a cube, which Malin immediately put back in place. As she did so, she gave Tim a reprimanding look (Kidwell 2005), and looked down again before Tim looked back at her. In other words, they did not make eye contact, but Tim saw Malin’s reprimanding look. Thus, they have just had a rather disaffiliative interaction. As the transcript begins, the robot vocalises an elongated “oh” with rising intonation, and starts moving towards Tim.

**EXTRACT 5:**
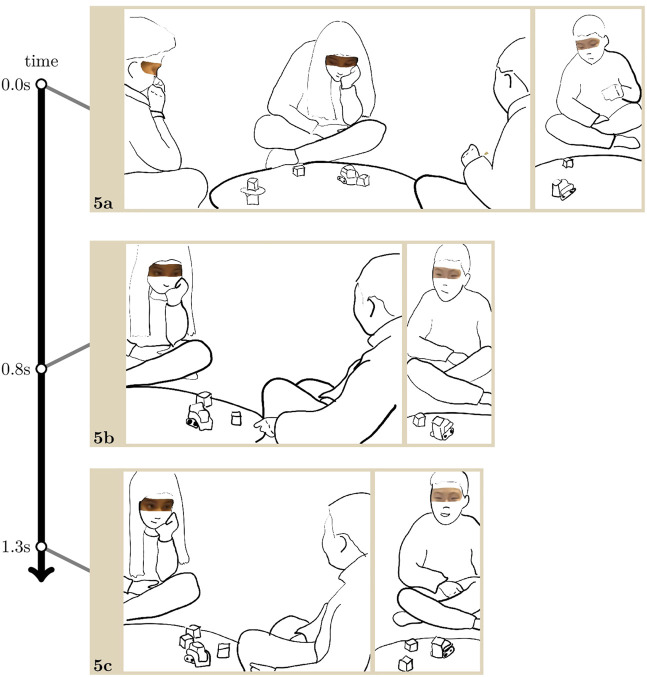


The robot pushes a cube on its way (Image 5. a) and slightly zigzags while moving towards Tim. Ben giggles with high-pitched, rapid sounds, and while he still appears as laughing by shaking his upper body, Tim looks at Malin (5. b). While Malin is following the robot’s movement with her gaze, she extends her head movement upward towards Tim in an immediate response to his look. Their eyes meet, Tim smiles, and Malin reciprocates the smile (Image 5. b). As one can expect, considering what studies on human gaze have largely established, they do not sustain this mutual gaze and look at the robot again while it is still moving towards Tim. This whole series of actions takes about 3 s only.

Like in the previous extract, Ben’s giggle contributes to creating a group dynamic and supports the subsequent interaction between Tim and Malin. As for the actions from the robot at the origins of Ben’s giggle and Malin’s and Tim’s mutual smiling gaze, its vocalisation on a questioning intonation, followed by wobbling towards Tim, they could be interpreted as an inquisitive attempt to approach Tim. Its movement also supports Malin’s gaze by somehow guiding it towards Tim: when Tim looks at her, she perceives it and simply extends the movement of her eyes. While simple in itself, the robot’s conduct is produced at a particular moment in the course of actions: shortly after Malin reprimanded Tim for making a wrong move with the cubes. It could be that in the aftermath, the children also take the first opportunity to revert the negative interactional dynamic into a positive one, and this simple conduct from the robot is a timely candidate. Indeed, by treating it as laughable, the children can interact in order to share emotional reactions. Albeit brief, the mutual gaze immediately followed by a smile between Malin and Tim is a highly affiliative interaction displaying a strong degree of intersubjectivity, especially between strangers, paving the way for long-term positive interactions at school.

For the design of social robot behaviour, this suggests that robots’ ability to generate emotional reactions may be sought not as much in the intrinsic form of their appearance or their actions, as in their ability to perceive transitioning relevant moments in the children’s interaction, and take action then. In Extract 5, the way the robot moves from Malin to Tim is key to their subsequent mutual gaze and smile. It encourages and mediates it right after a negative interaction, through its “funny” conduct and motion between the two children. In Extract 3, the robot could provide greater support by being capable of perceiving both the emotional reaction and the group dynamic in its offing as soon as Gil and Lisa turn to each other, and building on them by for example attempting to generate a similar reaction with the outgroup child so as to bridge the small gap between this outgroup and the ingroup children.

## Discussion

### Mediating Interaction Through Joint Orientation and Movements

Our initial observations and further analysis of the recorded interactions revealed how difficult and sensitive making eye contact can be for children who do not know each other and assume they have a limited shared background and no common language to communicate. By focusing on the emergence of interaction through gaze, we show how the robot makes co-presence possible, and provides a bedrock for interaction. Indeed, the children’s joint orientation to the robot—through gaze, but also in their body posture—constitutes a safe “home position” (Sacks and Schegloff 2002) to display engagement in the activity. From this home position, they can from time to time look at one another, and look away again any time. Additionally, we showed how the robot’s mobility and use of space supports gaze shifts, thus enables eye contact (Extract 5) and encourages interaction. While the first study showed with quantitative evidence that the robot’s behaviour makes the children reach into each other’s proximal space, this qualitative finding not only confirms the former but also shows in what sorts of situations and how it is accomplished.

### Distinguishing Collaboration and Personal Involvement in Interaction

We showed that instances of mutual gaze between unacquainted children occurred as part of two distinctive courses of actions and interactions in which children get more or less personally involved. When they ask for or give instructions, mutual gaze can be devoid of affective displays (typically showing a neutral face) and thereby little engaging with regards to interpersonal relationships. On the other hand, when they share an emotional reaction, gaze often goes along with a smile. As shown in Extract 5 where one child’s smile is reciprocated by its recipient, mutual, smiling gazes are elusive but positive, affiliative interactions which open the possibility for bonding. While unacquainted children may find it easier to initiate collaborative sequences to start with, sharing emotional displays is more likely to lead to relationship building. Therefore, this new understanding of the distinctive effects of task-oriented collaboration and interpersonal engagement in emotional reactions is particularly useful for the design of future HRI experiments which may want to include both.

### Group Dynamics

Two findings are particularly informative regarding group dynamics. Firstly, when they share an emotional reaction, the two ingroup, acquainted children can open up to and “invite” the outgroup, unacquainted child to join^5^ (Extract 3). Secondly, emotional reactions to the robot’s conduct from one of the ingroup children can create a propitious environment for the other two, unacquainted children to build on it and make contact to share this emotional reaction (Extracts 4, 5). In other words, the robot can encourage inclusion of the outgroup member by eliciting emotional reactions. The more sensitive robots will be to what elusive signs of tentative contacts between the participants, the more they will be able to build on these tentative contacts and support sustained interaction.

Furthermore, building on Strohkorb Sebo et al. (2020) and Fraune et al. (2019), we argue that evaluating a robot’s effects on group dynamics also requires studying interactions in which participants are concerned with the consequences of this isolated interaction on their personal relationship on a long term, instead of strictly experimental data where no relationship is at stake for them. In our data, that the children are in the same school and will meet again is integral to their hesitations and attempts to make positive contact, which could have been completely different were they not meant to see each other again. While HRI commonly relies on psychological approaches based on theories of the mind, by emphasising what is at stake for these children, we hope to have shown that more situated understandings of interpersonal, social processes are more relevant and useful to design social robots.

### The Robot as a Particular Entity

Our findings also shed new light on an important and yet surprisingly understudied topic in HRI: the sort of being or entity robots are addressed and treated as by humans. In our data, by reacting emotionally to the robot’s actions or apologising to the robot, the children treat it as an entity with a sense of self and moral rights, capable of (communicating) emotions and intentions—e.g., acting funny, being offended. Attribution of human-like features or capacities is central to human-robot interactions and to long-term relationship building. In our study, the unacquainted children often make contact as they react to the robot’s conduct as funny, laughable or “sweet”, “cute”, and all our cases of mutual smiling gaze occur in these trajectories. HRI has extensively studied which features of robot appearance and what kind of movements are the most influential for humans to empathise with robots (e.g., Philips et al., 2018; Venture and Dana, 2019). While this is not our focus, our findings emphasise the role of sharing emotions for unacquainted children to interact, presenting an opportunity for social robots to mediate interactions by eliciting emotions. Like animals and other non-human animated agents, robots in their variegated forms can foster powerful affective reactions when they are perceived as “cute” (Gn 2017). While there are concerns over the potential risks of emotional bonding with robots intentionally designed as cute (e.g., Lacey and Caudwell 2018) and the ethical problems it poses, as we shift from human-robot interactions to robot-mediated human-human interactions, cuteness becomes a more innocuous and even helpful tool.

### Different Effects of Robot Behaviour on Inclusion Processes

In their study, Sebo et al. (2020) attempted similar inclusion effects in groups of three participants comprising an outgroup member, playing a game. However, the robot provided verbal support, and while this was found effective in encouraging the outgroup member to participate, it also discharged ingroup members from making this sort of inclusive efforts. Thus, in their conclusions the authors ask whether the end effect is actually beneficial. Our findings show that less explicit actions from a social robot—non-verbal and suggestive actions, mainly movements and noises, indications on how to play the game, behaviours designed for being funny—can on the contrary encourage the ingroup members to take a step towards the outgroup member and make contact. The analysis of the automatic measures considering the adaptive robot behaviour in our first study showed that outgroup children were more outgoing in moving the cubes. The results of this work show that targeting the ingroup members and encouraging them to open to the outgroup members gives further opportunities to foster inclusion among children. Additionally, we want to argue that social robots are more likely to support inclusive behaviours by leaving part of the interactional work to the participants themselves, simply nudging them to do the rest of the work together through interactions. Indeed, it is *because* the instructions are partly incomplete or unclear that the unacquainted children make contact, to make sense of the instructions together (*Robot Requires to Take Action: Mutual Gaze as Part of Giving and Asking for Instructions*). Or, it is largely *because* the robot’s conduct is unexpected and subject to interpretation (unlike, say, a human-like, verbal utterance) that the children react vividly and come to share their emotional reaction [*The Robot’s Conduct Elicits Emotional Reactions: Looking and Smiling to the Other as Sharing Emotions* (*Towards Bonding*)]. In this way, we build on Sebo et al. (2020) and others’ endeavour to delineate the sort of robots and robot conduct likely to not only foster inclusion but foster inclusive behaviours in groups.

### Design Implications

The results inform both the design of HRI in group interactions, to foster and support positive group dynamics, and the design of social robots for the specific application scenario. We flag two critical capacities for designers to develop: that of perceiving mutual gazes between humans in groups, and that of understanding the more general interactional, social process each of these gazes can participate in. With the second phenomenon investigated, we argue that robots’ ability to generate emotional reactions may be sought not as much in the intrinsic form of their appearance or their actions, as in their ability to perceive transitioning, opportunistic moments in the children’s interaction, and then produce actions likely to generate emotional displays. While these design implications are difficult to achieve, probably a long way from today robots’ capacities and designers’ powers, we suggest they be considered as horizons, general but also reliable directions.

### A Guideline for Interdisciplinary Research

With this study, we tested a form of interdisciplinary research bringing together social robot design and research on interactions from the social sciences, around the use of video-recorded interactions as data. Initially, the two fields have contrastive methods and approaches to this data: while the first one is quantitative, measuring and computing amplitudes and frequency of actions to find general tendencies; the second is qualitative, “manually” analysing the interactions—without tools or machines—to understanding participants’ actions from their perspective and unpack phenomena and patterns.

From this successful experience, we propose a guideline for interdisciplinary research. The two approaches and analytic methods were applied sequentially: the study was first conducted in a HRI approach, from set-up to results; and in a second phase, the study was considered anew with the social-scientific approach. The interdisciplinary conversation thus occurred in mainly two phases: during takeover, for the social scientist to understand the data, research questions, and aims of the study; and after the second round of analyses, to discuss their result and how they resonated with those of the first study. We want to highlight several benefits of this collaboration. Firstly, for social-scientific research on interactions, the video data and the initial research questions were unique opportunities to investigate phenomena otherwise very difficult to capture “in the wild”, and the results build on research on gaze and children interactions. Secondly, the contrastive, qualitative approach revealed to robot designers aspects of the interactions which they could not grasp with their methods, and thus gave them a new understanding of the data and of what the robot was achieving. Third and lastly, to understand each other’s results and write together, we reached unprecedented (on both sides) levels of mutual understanding, with invaluable mutual learning as a result, which will influence, and already has influenced, our future research.

## Conclusion

The general aim of our study is to propose ways in which social robotics can help to foster inclusion of children who recently arrived in a country in the group of peers. The initial study was therefore designed to include elements that are known to help overcome intergroup bias such as the jigsaw method (Aronson, 2000). In this endeavour, we built a task that required contributions from each member of the group for the group to succeed, and we developed a robot behaviour that further ensured inclusion of the outgroup member. The robot’s behaviour and the conclusions drawn were based on an in-game measure which served as an abstraction for the dynamics present in the group. This abstraction was sufficient to understand the effect of the robot’s behaviour, but it could not unpack the complexity of the groups’ development and dynamics. To deepen the understanding of how this particular robot behaviour fosters the inclusion of newly arrived children, we decided to undertake video analysis in the perspective of social sciences, taking children’s perspective and considering their practical and relational problems during the experiments.

In this paper we presented the results from this second round of analysis. This approach revealed first off how difficult and delicate it is to make eye contact for children who do not know each other and assume that they have no common language, a limited shared background to communicate. We focused our analyses on the emergence of interaction through gaze.

In this way, this paper contributes to HRI research and any related fields using video-recorded interactions, by giving a glimpse of what *can* be investigated when we, researchers, look beyond the features defined in and as our initial research questions and protocol. Once the video data have been analysed within the initial framework (research questions, variables, hypotheses, etc.), there is a possibility to go beyond the pre-defined features, and see local interactional processes occurring in the live, video-captured episodes, worthy of further investigation. Our qualitative, fine-grained analyses of embodied interactions in actual instances reveal some of the utter complexities of human interactions in groups, emphasising participants’ perspective in otherwise highly controlled, experimental situations. Finally, we hope to encourage interdisciplinary research on human-(human-)robot interaction by showing its benefits and proposing a practical guideline.

## Data Availability

The data analyzed in this study is subject to the following licenses/restrictions: The data cannot be shared beyond the research group. The data must be anonymised before publication. Requests to access these datasets should be directed to; sgillet@kth.se.

## Footnotes

1 While a very rich area of research in itself, the different qualities of gazes and sequences of gaze crossing and averting are beyond the scope of this paper

2 The names are all pseudonyms. The images were transformed so as to preserve both the children’s anonymity and the visibility of relevant aspects of their faces

3 In Conversation Analysis, “preference” refers to social norms rather than personal inclinations. For example, acceptance is known as the “preferred” response to an invitation, and therefore rejections to invitations are done in specific ways marking “dispreference”, e.g., delay before responding, hesitation markers. Although we are not aware of studies relevant for this particular situation, Ali’s rejection of Andrej’s directive can be considered a “dispreferred” response, which a smile would be at odds with. See Pomerantz and Heritage (2013).

4 Even though this was the second time they were gathered for the experiment, the ingroup and the two ingroup children avoided mutual gaze and did not seem to communicate in any ways, just like in their first session together

5 Similar phenomena have been studied in multiparty human interactions, for example in the workplace (Tuncer 2018).
